# Training intervention effects on cognitive performance and neuronal plasticity—A pilot study

**DOI:** 10.3389/fneur.2022.773813

**Published:** 2022-08-05

**Authors:** Christine Wiebking, Chiao-I Lin, Pia-Maria Wippert

**Affiliations:** ^1^Medical Sociology and Psychobiology, University of Potsdam, Potsdam, Germany; ^2^Faculty of Health Sciences Brandenburg (Joint Faculty of the University of Potsdam, the Brandenburg Medical School Theodor Fontane, and the Brandenburg University of Technology Cottbus-Senftenberg), Cottbus, Germany

**Keywords:** chronic back pain, sensorimotor training intervention, multimodal intervention, MRI, neuroplasticity

## Abstract

Studies suggest that people suffering from chronic pain may have altered brain plasticity, along with altered functional connectivity between pain-processing brain regions. These may be related to decreased mood and cognitive performance. There is some debate as to whether physical activity combined with behavioral therapy (e.g. cognitive distraction, body scan) may counteract these changes. However, underlying neuronal mechanisms are unclear. The aim of the current pilot study with a 3-armed randomized controlled trial design was to examine the effects of sensorimotor training for nonspecific chronic low back pain on (1) cognitive performance; (2) fMRI activity co-fluctuations (functional connectivity) between pain-related brain regions; and (3) the relationship between functional connectivity and subjective variables (pain and depression). Six hundred and sixty two volunteers with non-specific chronic low back pain were randomly allocated to a unimodal (sensorimotor training), multidisciplinary (sensorimotor training and behavioral therapy) intervention, or to a control group within a multicenter study. A subsample of patients (*n* = 21) from one study center participated in the pilot study presented here. Measurements were at baseline, during (3 weeks, M2) and after intervention (12 weeks, M4 and 24 weeks, M5). Cognitive performance was measured by the Trail Making Test and functional connectivity by MRI. Pain perception and depression were assessed by the Von Korff questionnaire and the Hospital and Anxiety. Group differences were calculated by univariate and repeated ANOVA measures and Bayesian statistics; correlations by Pearson's r. Change and correlation of functional connection were analyzed within a pooled intervention group (uni-, multidisciplinary group). Results revealed that participants with increased pain intensity at baseline showed higher functional connectivity between pain-related brain areas used as ROIs in this study. Though small sample sizes limit generalization, cognitive performance increased in the multimodal group. Increased functional connectivity was observed in participants with increased pain ratings. Pain ratings and connectivity in pain-related brain regions decreased after the intervention. The results provide preliminary indication that intervention effects can potentially be achieved on the cognitive and neuronal level. The intervention may be suitable for therapy and prevention of non-specific chronic low back pain.

## Introduction

Chronic unspecific back pain is one of the most common chronic diseases in modern industrialized countries (1). It is a major risk factor for reduced time of physical activity, increased periods of inactivity, and for sick-leave, resulting in a significant reduction in quality-of-life and representing a major economic burden (2). Until now, it remains unclear which neurobiological mechanisms enable the transition from acute to chronic back pain. It is assumed that cortical reorganization processes and functional plasticity play a role in this process (3, 4).

Some studies report a reduction of both the thickness as well as the volume of gray matter in multiple brain regions [e.g., in the dorsolateral prefrontal cortex (3, 4), right anterior thalamus, brainstem, somatosensory cortex (3) and posterior parietal cortex (5)] in people with chronic unspecific back pain. Other research groups investigated whether chronic unspecific back pain could have an effect on the cortical representation of the back and found changes in the primary (S1) (6) as well as in the secondary (S2) (7) somatosensory cortex. In addition, Grachev and colleagues (8) showed that concentrations of brain metabolites were altered in patients with chronic back pain. The duration as well as the intensity of pain were related to neurotransmitters in different brain regions such as prefrontal areas, leading to patterns of abnormal chemical connectivity (8). For the transition from acute to chronic back pain corticostriatal connectivity seems to play an important role (9). The somatosensory cortex probably plays a significant role in pain perception and processing and therefore new therapy concepts are required that can influence this connection (10). Furthermore, the genesis of chronic pain is a complex and multidimensional process and involves more than neuroanatomical, functional and neurochemical changes. In a first step, neurochemical mediators seem to trigger reorganization processes in nociceptors and increase sensitivity for painful stimuli in the periphery (hyperalgesia). In a second step, these mediators seem to change central reciprocal pain inhibition (11), i.e., ascending pain pathways project to brain areas such as the somatosensory cortex, which in turn influence descending antinociceptive pathways from the brainstem. As such, significant changes of the neurochemical profile in brain areas such as the dorsolateral prefrontal cortex, thalamus and orbitofrontal cortex were observed in patients with chronic unspecific back pain (12).

Although the reciprocal pain regulation is well documented in the literature, our knowledge about the origin and formation of chronic pain syndromes and effective therapies is still limited (11, 13). On the one hand, practical approaches try to identify the inhibitor with the help of pharmaceutical solutions, for example through steroids, non-steroidal anti-inflammatory drugs, and serotonin or norepinephrine reuptake inhibitors. On the other hand, the behavioral and movement sciences have investigated the effects of physical activity and modulatory changes through cognitive tasks (14). Physical activity is associated with increased volume of gray and white matter (15) and increased blood supply in multiple brain regions (e.g., cerebellum, motor cortex, hippocampus, frontal cortex) (16, 17). This leads to the idea that physical activity may counteract chronic pain related changes. In any case, physical training is state of the art when it comes to the prevention and therapy of pain in patients with chronic low back pain (18). It aims to improve the efficiency of strength, sensorimotor functions of trunk muscles and neuromuscular control (19).

Whilst physical activity has effects on brain plasticity, several neuropsychological studies showed that the subjective perception of pain is closely related to attention and cognitive processes (20): pain is perceived with less intensity if subjects are distracted from their pain by the use of demanding cognitive tasks. Interestingly, stress also seems to interact with attention (21) as long lasting stress exposure is linked to altered neurotransmitter concentrations in brain regions relevant for the processing of pain. This in turn may have consequences for the transmission of pain signals and can have an influence on the information transfer in the sensorimotor system. In addition, patients suffering from severe chronic pain show decreased cognitive performance. Altogether, these results underline the need for developing novel multidimensional approaches, which investigate the relationship between physical activity, stress and distraction from subjective pain perception as well as underlying neuronal mechanisms. The presented pilot study, which was a part of the multicentre feasibility study of the National Research Network for Medicine in Spine Exercise [MiSpEx, www.mispex.de, Multicentre Study B MSB, further details see (22)], sought to shed light on these questions.

Though general inferences are restricted, the pilot study was used to test intended therapy effects in a sub-sample from the full MSB study. Moreover, the promising potential of future research projects shall be evaluated. The objectives of the pilot study are to analyse (1) the changes of cognitive performance over time and dependency on the intervention type, (2) the structural and functional changes in pain processing brain areas after intervention, and (3) the relationship between structural/functional changes in pain processing areas, subjective pain perception and depression.

## Materials and methods

### Design and procedure

After fulfilling the inclusion criteria, participants were randomly allocated to the different intervention groups (n_block_ = 18, basis 1:1; www.randomization.com). One group received a unimodal intervention with sensorimotor training (SMT), another a multidisciplinary intervention consisting of sensorimotor training with supporting behavioral therapy (SMT+BT) and a third group usual care (control group, CG). The sample size calculation of the MSB study was according to an unpublished dataset (a ≤ 0.05; 1-β = 0.999, drop out 30%, power analysis by G^*^Power,36 effect size *f* = 0.25, sample size: *n* = 600). The MSB study was registered as a clinical trial on 05/16/2013 in the German Clinical Trial Register with the identification number: DRKS00004977 (https://www.drks.de/drks_web/navigate.do?navigationId=trial.HTML&TRIAL_ID=DRKS00004977) (22–24). A subsample of MSB out of one study center (study center Potsdam) was used for the here presented pilot study.

Participants of the pilot study took part in the same measurement setup as in the MSB: they filled in questionnaires and underwent medical and biomechanical examinations at baseline, after 3 weeks (end of center-based intervention, M2), after 6 weeks (M3) and after 12 weeks (M4) (end of home-based intervention) and after 6 months (sustainability follow up M5) (22–24).

**Figure 1:**
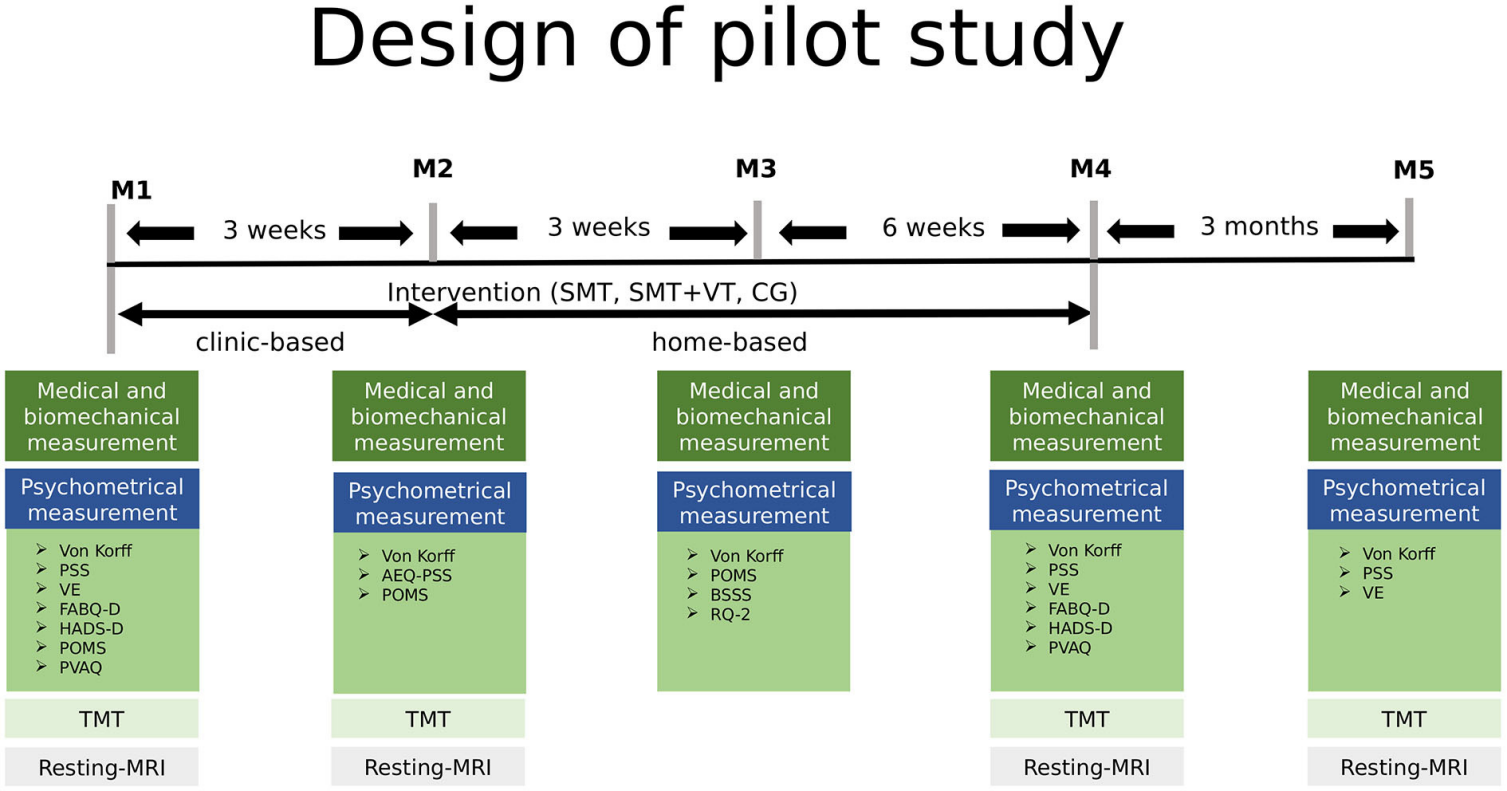
Study design of the current study including five measurement points baseline to M5 over a period of 6 months. MRI was measured at the FU Berlin, the remaining tests at the University of Potsdam, Germany. M2-M5, measurement 2-5; SMT, sensorimotor training; SMT-BT, sensorimotor training with behavioral therapy elements; CG, control group; PSS, German version of the Perceived Stress Scale; VE, German version of the Maastricht Vital Exhaustion Questionnaire–Short Form; FABQ-D, German version of the Fear Avoidance Beliefs Questionnaire; HADS-D, German version of the Hospital Anxiety and Depression Scale; POMS, German version of the Profile of Mood States; PVAQ, German version of the Pain Vigilance and Avoidance Questionnaire; TMT, Trail Making Test.

For the pilot study participants underwent magnetic resonance imaging (MRI) and Trail Making Tests (TMT) at baseline, M2, M4 and M5 (see Figure 1). The imaging data were recorded at the FU Berlin (Dahlem Institute for Neuroimaging of Emotion: Neurocomputation and Neuroimaging). No incidental findings occurred in the present study.

### Participants

In total, 744 persons were screened for MSB. Finally 662 were included. Of these 154 participants participated at study center Potsdam. Out of these, 21 were recruited for the pilot study. Participants were between 18 and 65 years old, suffered from intermittent low back pain, were fluent in German and able to fill out a questionnaire independently. The intermittent low back pain is a nonspecific low back pain event lasts four or more days in the past 12 months. Exclusion criteria were limited mobility, acute back pain within the last 7 days, implants or other metallic parts in/on the body (e.g., metallic contraceptive coil, bone screws, pacemaker, etc.,), pregnancy, pre-existing cardiovascular and psychological diseases/conditions, limited cognitive functions, tattoos, claustrophobia, diabetes and tinnitus. Participants were informed about the experiment at the time of recruitment and provided written informed consent prior to taking part in the study. The participation was voluntarily and was not compensated.

### Intervention

The detailed structure of the training program has been described in different MSB study publications from the MiSpEx network (23, 24). The training courses were carried out in a clinical environment (center-based, first 3 weeks) and at home-based (following 9 weeks). Each training session was 40 min. In the clinical phase, a sport- or physiotherapist guide the training sessions. During the home-based phase, participants trained with a DVD exemplifying the training and wrote a training dairy (22).

There were two intervention groups: the unimodal group (SMT) and the multidisciplinary group (SMT+BT). Within the unimodal group, SMT was applied, which comprised 12 weeks of training. In the first 3 weeks, guidance was offered in a study center three times a week for 30 min. The training continued for 9 weeks at home and was supported by a DVD exemplifying the exercises. The training consisted of four different exercises (stability, deadlift/rowing, heel stand, and side planks). Within the meaning *graded activity*, the exercises can be adjusted to 12 different levels of difficulty in order to allow individual increases intensity (22).

Within the multidisciplinary group, the sensorimotor training was supported by behavioral therapy (SMT+BT), which consisted of cognitive distraction during the SMT exercises, psychoeducation, and a body-scan. Cognitive distraction effectively influences pain inhibition and may relate to decreasing fear-avoidance to exercise intervention (23, 25, 26). The participants of this group were cognitively distracted during the SMT training by n-back tasks. N-back tasks were performed at different levels. Participants performed 1-back at week 1–3, 2-back at week 4–7, and 3-back at week 7–12. The distraction task was guided by a DVD and performed while exercising. Exercise repetitions were timely adapted to cognitive tasks.

At the end of each training session, the participants performed a body scan, which was based on the *Mindfulness Based Stress Reduction* programme. This is complemented by a detailed patient education (including partner, if applicable) providing knowledge about the origin and maintenance of pain as well as goal-orientated coping strategies in everyday life [for detailed design and intended effects see (22)]. The CG receive usual care.

### Instruments and methods

#### Pain, stress, and mental health

Pain intensity and subjective pain disability were investigated using the Von Korff questionnaire (27). Stress was assessed by psychometric instruments for the following constructs: subjective stress *via* the Perceived Stress Scale (PSS) (28), vital exhaustion *via* the Maastricht Vital Exhaustion Questionnaire – Short Form (VE) (29) and life events (LE) by singular items; the mental health section included: for pain/emotion processing the Fear-Avoidance-Beliefs-Questionnaire (FABQ-D) (30), Avoidance-Endurance-Questionnaire (AEQ-PPS) (31) and the Pain Vigilance and Avoidance Questionnaire (PVAQ) (32); for anxiety and depression the Hospital Anxiety and Depression Scale (HADS-D) (33), and for Mood the Profile of Mood States (POMS) (34). Aspects of personal life and (health) care *via* sociodemographic information, the Berlin Social Support Scales (BSSS) (35) and the Relationship Questionnaire (RQ-2) (36). The use of medication and stimulants was documented adjacent to sleeping habits.

#### Cognitive performance

As chronic pain is associated with slower psychomotor and processing speed (37, 38), the TMT was used. The TMT is a test commonly used in behavioral research to investigate visuomotor processing speed, executive functions and attention of neuropsychological functions (e.g., cognitive flexibility and working memory) (39). The TMT was chosen because of the great test-retest reliability of the TMT-A (0.76 and 0.89) and its feasibility in mobile studies (40). By using a touch-sensitive tablet to record TMT test results, measurements can be made more mobile. This is of advantage in clinical studies (“from bench to bedside”) or in home-based study designs. As the current pilot study was conducted to test the feasibility and evaluate the potential of future research projects in a first step, further developments in mobile and more autonomous environments show promising perspectives.

**Figure 2:**
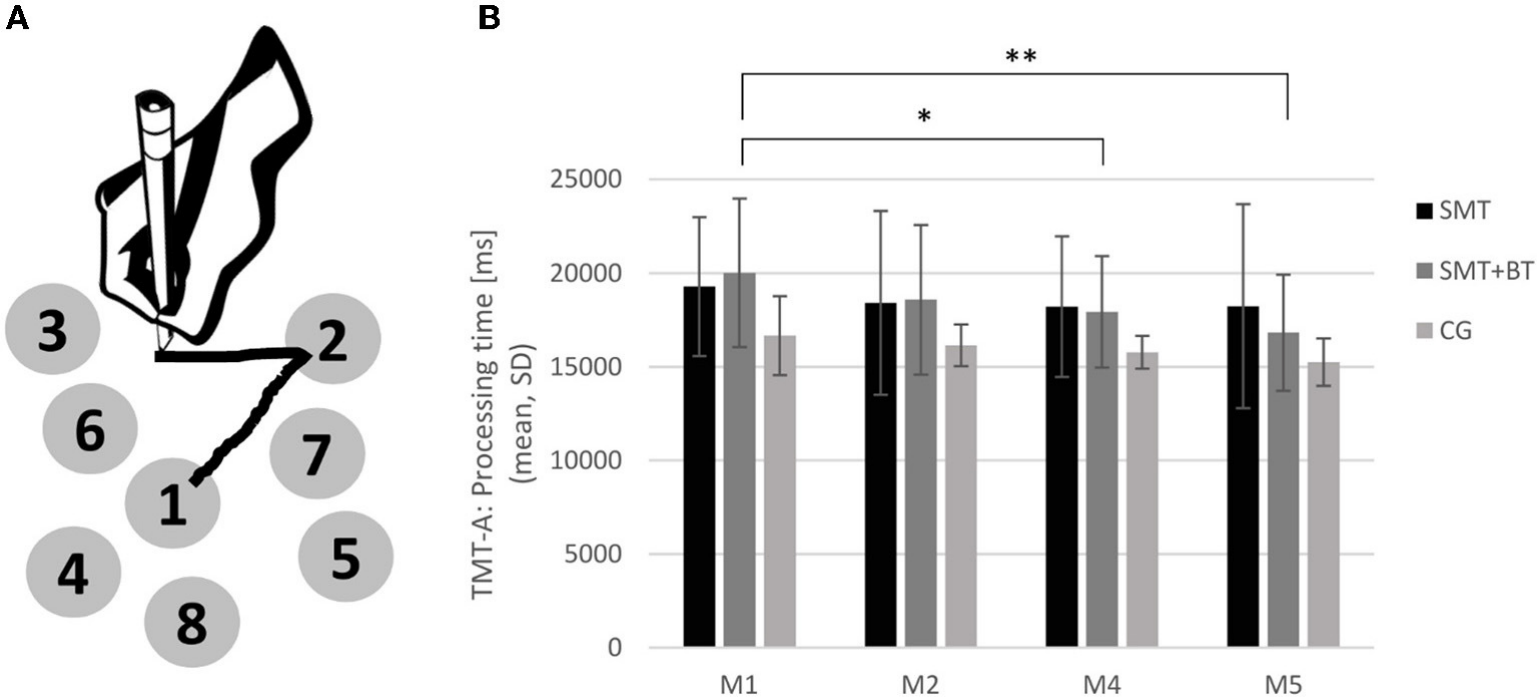
**(A)** Illustration of the Trail Making Test (TMT-A) and the development of cognitive performance; **(B)** The performance of TMT-A across the different measurement points baseline, M2, M4, and M5 for each group separately (SMT, sensorimotor training; SMT+BT, sensorimotor training with behavioral therapy elements; CG, control group). The multidisciplinary training group (SMT + BT: *n* = 5) improved after 3 (M4) and 6 month (M5). *p < *0.0*5 **p < *0.0*1.

In more detail, the TMT-A (see Figure 2A) measures predominantly processing speed, whereas part B captures higher cognitive performance (e.g., cognitive flexibility). Part A consisted of pseudo-randomly distributed numbered circles ranging from 1 to 25. Subjects were asked to connect the circled numbers in an ascending order as quickly as possible with high accuracy. The time to complete the TMT was measured accordingly and mirrors processing speed and cognitive flexibility. The TMT was presented using the software PEBL (Psychology Experiment Building Language) on a DELL laptop with a standard PnP-monitor (32-bit).

#### MRI data acquisition

MRI scans were acquired on a 3-Tesla whole body system (Siemens Trio, Erlangen, Germany) equipped with a 32-channel head coil. Each scan consisted of sequences for structural MRI (T_1_, MPRAGE, spatial resolution 1 x 1 x 1 mm^3^; echo time 2.52 ms; repetition time 1900 ms; flip angle 9°) and functional recordings in resting-state (${\text{T}}_{2}^{*}$, echo planar images, spatial resolution 3 x 3 x 3.2 mm^3^, echo time 30 ms; repetition time 2300 ms; flip angle 70°). The whole-brain ${\text{T}}_{2}^{*}$-weighted echo planar images consisted of 37 slices per volume. Slices were aligned with the AC-PC line (anterior and posterior commissure). Functional resting-state MRI (rsMRI) scans consisted of 160 volumes per participant. During the acquisition of rsMRI and structural MRI, the participants were asked to open their eyes and a fixation cross was projected onto a screen visible through a mirror mounted on the headcoil.

### Statistical analysis

The standardized psychometric tests were prepared according to the accompanying manuals.

#### Cognitive performance

The results of the TMT were calculated as ratio and difference scores between processing time and errors. The data of the TMT measurements were analyzed descriptively and inferentially. Outlier were excluded in order to avoid skewed distributions and distorted results driven by single outlier values. Outliers were recognized by the boxplot from SPSS (outliers are defined as values which is beyond the range between 3rd quartile + 1.5^*^interquartile range and 1^st^ quartile −1.5^*^interquartile range). A one-way ANOVA and Bayesian one-way ANOVA were employed to assess group differences before intervention. Repeated measures ANOVAs and Bayesian repeated measures ANOVA were applied to investigate differences in cognitive performance after 3 weeks and 3 months of intervention within the groups. Statistical significance was set to *p* < *0.0*5 and *p* < *0.0*17 for multiple tests (applying a Bonferroni correction). For interpretation of Bayes factors, see Andraszewicz et al. (41). Statistical analyses were performed using IBM SPSS Statistics Version 20 (SPSS Inc., Chicago, IL) and by JASP (Version 0.16) [Windows] for Bayesian statistics.

#### Processing of MRI data

MRI data from 20 participants were preprocessed and statistically analyzed using the software FSL (release revision 5.0.8, see https://fsl.fmrib.ox.ac.uk/fsl/fslwiki/) (42, 43). Functional images were corrected for head movement [MCFLIRT, (44)], motion outliers (see FSL Motion Outliers), brain-extracted [Brain Extraction Tool, (45)], high-pass filtered (100 s) and smoothed with a 5 mm Gaussian filter. Furthermore, an independent component analysis [ICA, MELODIC Toolbox (46)] was performed, and data were divided into 25 statistically independent components from which noisy components were removed by using an operationalized procedure (47). Individual time series of affected components were classified as artifacts and excluded from the entire data set by linear regression. In more detail, 13 components from the baseline measurement, M4 and M5 were removed, respectively. 15 components were removed from M2. Finally, anatomical brain regions of interest (ROI) were defined based on Cifre et al. (48) (please refer to Supplementary material): the periaqueductal gray (PAG), the primary motor cortex (M1), the primary/secondary somatosensory area (S1/S2) and the supplementary motor area (SMA). Functional connectivity analyses, based on the temporal signal course between ROIs, were performed using the Z-transformed correlation coefficients in Python 2.7.6.

T_1_-images were statistically analyzed according to the modified VBM protocol for longitudinal data (49, 50). Images were brain-extracted, segmented (white matter, cerebrospinal fluid, gray matter) and smoothed. The resulting gray matter images were non-linearly aligned to a study-specific template and modulated, i.e., each voxel of each registered gray matter image was divided by the Jacobian of the warp field in order to compensate for the contraction/enlargement of the non-linear component of the transformation (49). In order to control for gray matter in each ROI, the proportion of gray matter was calculated from gray matter images using the FSL FAST tool (http://fsl.fmrib.ox.ac.uk/) and included as a control variable in correlational ROI to ROI analyses (see Correlation between functional connectivity and depression).

#### Correlation between functional connectivity and depression

For this analysis, unimodal (SMT) and multidisciplinary (SMT+BT) were pooled as the intervention group (IG) while analyzing the change and correlation of functional connection because of the small sample size. The correlation between the changes of functional connectivity in pain processing areas (PAG and SMA) with the subscale depression of the HADS-D was investigated using Pearson's *r* (controlled for the volume of the gray matter in PAG and SMA). Statistical significance was set to *p* < *0.0*5.

## Results

### Descriptive of pain, stress and mental health

Twenty-one participants (age range between 20–62 years, *n* = 14 female) took part in the study. Nine participants were allocated to the multidisciplinary group (SMT), five to the multidisciplinary group (SMT+BT) and seven to the CG. During MRI measurements no incidental findings occurred and no participant was excluded due to excessive head motion. However, one subject was excluded and did not complete all examinations (*n* = 20 participants in MRI). Seven participants were smokers and consumed alcohol regularly (33.3%). 16 Participants exercised regularly (76.2%), whilst the average training time of the sample was 3.1 h per week (see Table 1).

**Table 1:** Descriptive information about the sample and psychometric information (left side) including ranges of the questionnaires.

		**SMT**				**SMT**+**BT**				**CG**			
		**Baseline** **(*n =* 9)**		**M4** ***(n =* 8)**		**Baseline** ***(n =* 5)**		**M4** ***(n =* 5)**		**Baseline** ***(n =* 7)**		**M4** ***(n =* 6)**	
		**M**	**SD**	**M**	**SD**	**M**	**SD**	**M**	**SD**	**M**	**SD**	**M**	**SD**
	Age (years)	35.3	11.3			44.2	12.9			31.3	9.1		
	Physical activity/week (hours)	2.3	1.7			2.5	0.7			4.0	4.9		
Pain	v. Korff Pain intensity (0–100)^a^	48.5	15.6	35.8	19.6	37.3	24.1	17.3	17.5	19.5	14.1	21.1	22.5
	v. Korff Pain disability (0–100)^a^	25.9	23.3	17.9	18.6	20.0	27.5	3.3	5.8	7.6	9.0	5.0	5.9
*Mental health*	HADS Anxiety (0–21)^d^	7.4	4.3	7.0	3.2	3.4	2.0	3.4	1.5	5.4	2.2	4.5	3.2
	HADS Depression (0–21)^d^	6.0	3.4	3.4	3.4	5.2	3.0	4.8	4.6	3.6	2.5	3.2	3.4
	POMS Depression/Anxiety (0–84)^e^	18.7	16.2			8.6	8.7			12.6	7.9		
	POMS Vigor (0–42)^e^	21.9	6.6			21.4	8.6			23.0	6.6		
	POMS Fatigue (0–42)^e^	19.1	9.3			12.6	10.6			16.4	6.7		
	POMS Hostility (0–42)^e^	10.4	7.2			3.4	3.1			7.7	7.7		
*Pain related cognition*	FABQ-D Relationship between back pain and physical activity (0–30)^b^	16.1	5.9	14.0	6.5	10.2	5.9	15.4	5.0	13.3	4.9	14.0	4.1
	FABQ-D Cause of back pain was work (0–30)^b^	12.0	7.7	12.5	6.1	6.6	5.9	4.8	5.5	5.1	6.0	4.2	6.2
	FABQ-D Forecasting of return to work (0–30)^b^	1.6	3.1	1.1	2.0	1.2	2.7	2.8	6.3	0.1	0.4	0.0	0.0
	PVAQ Pain attention (0–80)^c^	40.2	10.2	33.1	9.3	34.6	12.0	33.2	11.0	38.3	15.0	36.8	13.9
*Stress*	VE Vital exhaustion (0–18)^f^	8.9	4.9	6.6	5.8	7.0	5.7	7.0	4.4	6.4	5.0	6.8	5.7
	PSS Perceived Stress (1–40)^g^	18.8	4.8	16.6	5.0	14.6	3.7	15.2	1.9	18.6	5.4	17.3	6.2

Values show means (M) and standard deviation (SD). Results are shown for the three groups of the feasibility study: unimodal group (SMT), multidisciplinary group (SMT+BT) and control group (CG). M4 indicates the measurement after the intervention.

a The Von Korff pain questionnaire (CPI, DISS).

b Fear Avoidance Beliefs Questionnaire (FABQ-D).

c Pain Vigilance and Avoidance Questionnaire (PVAQ).

d Hospital Anxiety and Depression Scale (HADS-D).

e Profile of Mood States (POMS).

f Maastricht Vital Exhaustion Questionnaire—Short Form (VE).

g Perceived Stress Scale (PSS).

The number of participants taking painkillers showed no difference among three groups at baseline [X^2^(2) = 4.4, *p* = 0.11], measurement 4 [M4) (X^2^(2) = 1.45, *p* = 0.48] and measurement 5 (M5) [X^2^(2) = 0.78, *p* = 0.68]. Five participants took painkillers (Ibuprofen) at baseline, M4, and M5 (baseline: two participants from the control group; at M4, two from the control group, one from the SMT and one from SMT+BT; at M5 one from the SMT group). Apart from painkillers, some participants took birth control pills, daily supplements, allergy or thyroid medicine, potassium iodide, methotrexate, cold medicine, blood pressure control medicine or eye-drops.

### Changes in cognitive performance

Three outliers were excluded from data analysis [one from the CG at baseline measurement, one from the CG at M3, and one from the IG (SMT) at M4]. At the beginning of the measurements (baseline), the participants of the control group showed slightly better cognitive performance data compared to the intervention groups. However, this baseline difference did not reach statistical significance [CG vs. SMT: *F*(1, 14) = 2.36, *p* = *0.1*5; CG vs. SMT+BT: *F*(1, 11) = 3.02, *p* = *0.1*1; SMT vs. SMT+BT: *F*(1, 12) = 0.10, *p* = *0.7*6; Bayes factor = 0.90].

Considering the cognitive performance over time within the groups, the multidisciplinary intervention group (SMT+BT) showed significant improvements after 3 months (baseline vs. M4: *F*(1, 4) = 10.84, *p* = *0.0*3) and also after 6 months [baseline vs. M5 for TMT-A: *F*(1, 4) = 26.29, *p* < *0.0*1; see Figure 2B] with strong evidence (BF_10_ = 16.05, baseline vs. M4: BF_10_ = 3.19, baseline vs. M5: BF_10_ = 9.27); such a result could not be established for either the unimodal intervention group (SMT) or the CG [CG: baseline vs. M2: *F*(1, 6) = *0.53, p* = *0.5*0; baseline vs. M4: *F*(1, 6) = 2.14, *p* = *0.1*9; SMT-group: baseline vs. M2: *F*(1, 7) = 1.72, *p* = *0.2*3; baseline vs. M4: *F*(1, 7) = 1.60, *p* = *0.2*5]. The Bayes factor also indicates that the CG and SMT showed little change with time (anecdotal evidence, Bayes factor = 0.88 and 0.29).

### Changes of functional connectivity as a consequence of the intervention in pain processing brain areas

In order to investigate unbiased connectivity results between time points, the total sample was used to compare values (Table 2, left column). All ROIs showed a global decrease of connectivity between pre and post-intervention. Next, subgroups were established and connected to subjective pain ratings. In the IG, the central importance of the PAG could be underlined for processes of pain perception. Specifically, the region of interest in the PAG showed significant functional connectivity to the primary motor cortex (M1) and the precuneus. Moreover, the PAG-associated connectivity (post-intervention) was related to subjective pain intensity in the IG. Considering the CG, PAG-associated connectivity was not connected to subjective pain intensity (both pre- and post-intervention). Only in the IG there was a correlation between PAG-associated connectivity (post-intervention) and subjective pain intensity (see Table 2).

**Table 2:** Functional connectivity and connections between pain-relevant networks (ROIs) and subjective data; t-tests and correlations according to Pearson (r), p < 0.05.

	**Connectivity**		**Connectivity and correlation**			
	**Baseline—M4** **total sample**		**To pain intensity^a^ post-intervention**		**To depression^b^ post-intervention**	
	***t*-Test**		**IG**	**CG**	**IG**	**CG**
PAG-M1	*p < 0.0*01	pre > post	*r = 0.6*0, *p = 0.0*5	*n.s*.	*n.s*.	*n.s*.
PAG-S1	*p < 0.0*01	pre > post	*r = 0.6*5, *p = 0.0*3	*n.s*.	*n.s*.	*n.s*.
PAG-S2	*p < 0.0*01	pre > post	*r = 0.6*3, *p = 0.0*4	*n.s*.	*n.s*.	*n.s*.
PAG-SMA	*p < 0.0*01	pre> post	*r = 0.72, p = 0.01*	*n.s*.	*r = 0.69, p = 0.04*	*n.s*.

Intervention groups (IG): n = 13, control group (CG): n = 7.

M1, primary motor cortex (motor control); PAG, periaqueductal gray (pain processing), brain stem; S1, primary somatosensory area (representation of contralateral body surface); S2, secondary somatosensory area (bilateral representation); SMA, supplementary motor area (planning, selection of learned, non-stimulus-induced movement).

a Pain intensity was measured using the Von Korff pain questionnaire (0–100).

b Depression was measured by Hospital Anxiety and Depression Scale (HADS-D) (0–21).

### Correlation between functional connectivity and depression

The IG showed a correlation after the intervention (*r* = *0.6*91, *p* = *0.0*39). This relationship was not seen prior to the intervention (in neither the IG nor the CG) and could not be observed in other PAG-related areas (Table 2). No associations were observed following training on changes in pain and depression.

## Discussion

The current study investigated the effects of uni- and multidisciplinary sensorimotor training in combination with a cognitive distraction task on cognitive performance. Moreover, MRI was applied in order to investigate changes in functional connectivity and its association with pain processing in brain areas involved in pain processing.

The study results showed that cognitive performance, operationalized by scores of processing speed, increased over time in participants receiving multidisciplinary training (SMT+BT). This suggests that working memory tasks activating cognitive activities while exercising can serve as competing stimuli in pain related areas, finally leading to an improvement in cognitive performance (51–53). Further, this type of intervention may reduce cognitive impairment due to chronic pain, which can improve life quality and may be valuable for elderly patients (54). With that in mind, this training type may be a suitable tool for the prevention and therapy of chronic pain.

With regard to the relationship between functional changes in pain processing brain areas and pain perception, the results showed that the reduction of subjective pain intensity in the intervention group was correlated with reduced functional connectivity between PAG and sensory/motor areas. In addition to reduced subjective pain intensity, it seems that the involvement of functional connectivity between PAG and sensory/motor areas decreases continuously over time (55), which is only a global and unspecific effect that could be seen in the current results. Results related to pain intensity results are in line with study results of Buhle and Wager (20, 56), who showed that increased activity in the PAG is correlated with pain perception in healthy participants. Thus, the results lead to the assumption that the intervention programme potentially influences subjective pain intensity and the level of PAG-related neuronal activity. The relationship between functional changes in the PAG and SMA suggests that participants of the intervention could benefit from the training.

Moreover, the relationship between depressive mood and structural changes was confirmed in preliminary analyses of the current data set (57), as those subjects showed indeed a reduction of the gray matter in the brain [e.g. (58)]. The exact implications of this finding and its relevance in combination with physical activity, pain perception or possible interacting effects of training were not investigated. These questions need to be targeted in future research studies.

### Limitation

The current study can be classified as a pilot study that was used to investigate effects of a specific combined physical and behavioral training, which intended an erase of pain traces as well as an increase of neuromuscular control and cognitive performance (13). The current results may provide useful initial evidence for the development of new therapies for people suffering from centralized chronic pain problems, delayed reaction or motivation to start exercising and who had strong problems start exercising. Of course, the basic statistical analyses due to the low number of participants prevent further evaluations and statistical approaches, such as an interaction of group by time. As such, any inferences that can be drawn from the results about specific intervention effects are restricted. The results of the current pilot study, including small sample sizes, need to be interpreted with caution. Aiming at a better understanding of the complex interactions between physical training and pain processing, future studies need to increase sample sizes. In addition, as the TMT represents only a part of the bigger construct of cognitive performance, current results should be seen limited to performance speed. Finally, measurements of neurotransmitter concentrations *via* magnetic resonance spectroscopy can be used to further shed light on these complex relationships.

## Conclusion

The results of the current pilot study need to be considered with caution, as sample sizes are low and subsequent statistical analyses limited. However, the study design may serve as a good example for future studies investigating such intervention programs while targeting possible effects on brain and behavior. The present results suggest that changes on the behavioral and neuronal level may come into effect. Hence, these changes can be measured with well-considered instruments. In future studies the application of specific intervention programs, as described in the current pilot study, may be considered for application in therapy and prevention programs.

## Data availability statement

The raw data supporting the conclusions of this article will be made available by the authors, without undue reservation.

## Ethics statement

The research project was conducted in accordance with the Declaration of Helsinki and was approved by the Ethics Committee of the University of Potsdam, Germany. All participants gave their written informed consent before participating in this study.

## Author contributions

CW and P-MW: conceptualization. CW: methodology, software, data curation, formal analysis, and writing—original draft. C-IL: visualization. P-MW: principal investigator, project administration, supervision, and funding acquisition. CW, C-IL, and P-MW: writing—review and editing. All authors contributed to the article and approved the submitted version.

## Funding

This present study was funded by the German Federal Institute of Sport Science on behalf of the Federal Ministry of the Interior of Germany as the major funder. It is realized within MiSpEx—the National Research Network for Medicine in Spine Exercise (Grant Number: 080102A/11-14). The funder does not influence data collection, analysis, and interpretation or writing of the manuscript. We acknowledge the support of the Deutsche Forschungsgemeinschaft (DFG, German Research Foundation) and Open Access Publication Fund of the University of Potsdam (491466077).

## Conflict of interest

The authors declare that the research was conducted in the absence of any commercial or financial relationships that could be construed as a potential conflict of interest.

## Publisher's note

All claims expressed in this article are solely those of the authors and do not necessarily represent those of their affiliated organizations, or those of the publisher, the editors and the reviewers. Any product that may be evaluated in this article, or claim that may be made by its manufacturer, is not guaranteed or endorsed by the publisher.

## Abbreviations

CG: Control group
CPI: Characteristic Pain intensity, scale of the Von Korff pain questionnaire in German
DISS: subjective pain disability, scale of the Von Korff pain questionnaire in German
FABQ-D: German version of the Fear Avoidance Beliefs Questionnaire
HADS-D: German version of the Hospital Anxiety and Depression Scale
IG: intervention group
M1: primary motor cortex
M2, M3, M4, M5: visits 2–5
MiSpEx: the German Research Network of Medicine in Spine Exercise
MRI: magnetic resonance imaging
ROI: brain regions of interest
rsMRI: resting-state magnetic resonance imaging
N: number of participants
p -values: significance level: *p* < 0.01, *p* < 0.05 or *p* < 0.10
POMS: German version of the Profile of Mood States
PSS: German version of the Perceived Stress Scale
PVAQ: German version of the Pain Vigilance and Avoidance Questionnaire
PAG: periaqueductal gray
S1: primary somatosensory area
S2: secondary somatosensory area
SMA: supplementary motor area
SMT: sensorimotor training
SMT+BT: sensorimotor training with behavioral therapy elements
TMT: the Trail Making Test
VE: German version of the Maastricht Vital Exhaustion Questionnaire – Short Form.

## References

[1] Hoy D Bain C Williams G March L Brooks P Blyth F. A systematic review of the global prevalence of low back pain. Arthritis Rheum. (2012) 64:2028–37. 10.1002/art.34347 22231424

[2] Hoy D Brooks P Blyth F Buchbinder R. The Epidemiology of low back pain. Best Pract Res Clin Rheumatol. (2010) 24:769–81. 10.1016/j.berh.2010.10.002 21665125

[3] Apkarian AV Sosa Y Sonty S Levy RM Harden RN Parrish TB. Chronic back pain is associated with decreased prefrontal and thalamic gray matter density. J Neurosci. (2004) 24:10410–5. 10.1523/JNEUROSCI.2541-04.2004 15548656 PMC6730296

[4] Schmidt-Wilcke T Leinisch E Gänbauer S Draganski B Bogdahn U Altmeppen J. Affective components and intensity of pain correlate with structural differences in gray matter in chronic back pain patients. Pain. (2006) 125:89–97. 10.1016/j.pain.2006.05.004 16750298

[5] Buckalew N Haut MW Morrow L Weiner D. Chronic pain is associated with brain volume loss in older adults: preliminary evidence. Pain Med. (2008) 9:240–8. 10.1111/j.1526-4637.2008.00412.x 18298708

[6] Flor H Braun C Elbert T Birbaumer N. Extensive reorganization of primary somatosensory cortex in chronic back pain patients. Neurosci Lett. (1997) 224:5–8. 10.1016/S0304-3940(97)13441-3 9132689

[7] Hotz-Boendermaker S Marcar VL Meier ML Boendermaker B Humphreys BK. Reorganization in secondary somatosensory cortex in chronic low back pain patients. Spine. (2016) 41. 10.1097/BRS.0000000000001348 27244113

[8] Grachev ID Fredrickson BE Apkarian VA. Abnormal brain chemistry in chronic back pain: an in vivo proton magnetic resonance spectroscopy study. Pain. (2000) 89. 10.1016/S0304-3959(00)00340-7 11113288

[9] Baliki MN Petre B Torbey S Herrmann KM Huang L Schnitzer TJ. Corticostriatal functional connectivity predicts transition to chronic back pain. Nat Neurosci. (2012) 15:1117–9. 10.1038/nn.3153 22751038 PMC3411898

[10] Paulus MP. Neural basis of mindfulness interventions that moderate the impact of stress on the brain. Neuropsychopharmacology. (2016) 41:373–373. 10.1038/npp.2015.239 26657952 PMC4677133

[11] Harvey V Dickenson A. Neurobiology of pain. In: Stannard C Kalso E Ballantyne J, editors. Evidence-Based Chronic Pain Management. Oxford: Wiley-Blackwell (2010). p. 42–51.

[12] Wand BM Parkitny L O'connell NE Luomajoki H Mcauley JH Thacker M. Cortical changes in chronic low back pain: current state of the art and implications for clinical practice. Man Ther. (2011) 16:15–20. 10.1016/j.math.2010.06.008 20655796

[13] Wippert PM Wiebking C. Stress and alterations in the pain matrix: a biopsychosocial perspective on back pain and its prevention and treatment. Int J Environ Res Public Health. (2018) 15:785. 10.3390/ijerph15040785 29670003 PMC5923827

[14] Wippert PM Wiebking C. Adaptation an körperliche Aktivität und psychischen Stress im Kontext von Schmerz. Der Schmerz. (2016) 30:429–36. 10.1007/s00482-016-0147-0 27492492

[15] Erickson KI Gildengers AG Butters MA. Physical activity and brain plasticity in late adulthood. Dialogues Clin Neurosci. (2013) 15:99–108. 10.31887/DCNS.2013.15.1/kerickson 23576893 PMC3622473

[16] Kerr AL Steuer EL Pochtarev V Swain RA. Angiogenesis but not neurogenesis is critical for normal learning and memory acquisition. Neuroscience. (2010) 171:214–26. 10.1016/j.neuroscience.2010.08.008 20804819

[17] Vivar C Potter MC Van Praag H. All about running: synaptic plasticity, growth factors and adult hippocampal neurogenesis. Curr Top Behav Neurosci. (2013) 15:189–210. 10.1007/7854_2012_220 22847651 PMC4565722

[18] Hayden J Van Tulder MW Malmivaara A Koes BW. Exercise therapy for treatment of non-specific low back pain. Cochrane Database Syst Rev. (2005) 3:CD000335. 10.1002/14651858.CD000335.pub2 16034851 PMC10068907

[19] Moreno Catalá M Schroll A Laube G Arampatzis A. Muscle strength and neuromuscular control in low-back pain: elite athletes versus general population. Front Neurosci. (2018) 12:436–6. 10.3389/fnins.2018.00436 30018531 PMC6037821

[20] Buhle J Wager TD. Performance-dependent inhibition of pain by an executive working memory task. Pain. (2010) 149:19–26. 10.1016/j.pain.2009.10.027 20129735 PMC4229048

[21] Plessow F Kiesel A Kirschbaum C. The stressed prefrontal cortex and goal-directed behaviour: acute psychosocial stress impairs the flexible implementation of task goals. Exp Brain Res. (2012) 216:397–408. 10.1007/s00221-011-2943-1 22101494

[22] Wippert PM De Witt Huberts J Klipker K Gantz S Schiltenwolf M Mayer F. Beschreibung und empirische Fundierung des verhaltenstherapeutischen Moduls der MiSpEx-Intervention. Der Schmerz. (2015) 29:658–63. 10.1007/s00482-015-0044-y 26337688

[23] Wippert P-M Drießlein D Beck H Schneider C Puschmann A-K Banzer W. The feasibility and effectiveness of a new practical multidisciplinary treatment for low-back pain: a randomized controlled trial. J Clin Med. (2020) 9:115. 10.3390/jcm9010115 31906224 PMC7019545

[24] Wippert P-M Puschmann A-K Drießlein D Banzer W Beck H Schiltenwolf M. Personalized treatment suggestions: the validity and applicability of the risk-prevention-index social in low back pain exercise treatments. J Clin Med. (2020) 9:1197. 10.3390/jcm9041197 32331301 PMC7230931

[25] Verhoeven K Crombez G Eccleston C Van Ryckeghem DML Morley S Van Damme S. The role of motivation in distracting attention away from pain: An experimental study. Pain. (2010) 149:229–34. 10.1016/j.pain.2010.01.019 20188469

[26] Deldar Z Rustamov N Bois S Blanchette I Piché M. Enhancement of pain inhibition by working memory with anodal transcranial direct current stimulation of the left dorsolateral prefrontal cortex. J Physiol Sci. (2018) 68:825–36. 10.1007/s12576-018-0598-4 29450801 PMC10717442

[27] Von Korff M Ormel J Keefe FJ Dworkin SF. Grading the severity of chronic pain. Pain. (1992) 50:133–49. 10.1016/0304-3959(92)90154-4 1408309

[28] Cohen S Kamarck T Mermelstein R. A global measure of perceived stress. J Health Soc Behav. (1983) 24:385–96. 10.2307/2136404 6668417

[29] Appels A Hoppener P Mulder P. A questionnaire to assess premonitory symptoms of myocardial infarction. Int J Cardiol. (1987) 17:15–24. 10.1016/0167-5273(87)90029-5 3666994

[30] Pfingsten M Leibing E Franz C Bansemer D Busch O Hildebrandt J. Erfassung der “fear-avoidance-beliefs” bei Patienten mit Rückenschmerzen. Der Schmerz. (1997) 11:387–95. 10.1007/s004820050114 12799796

[31] Hasenbring MI Hallner D Rusu AC. Fear-avoidance- and endurance-related responses to pain: development and validation of the Avoidance-Endurance Questionnaire (AEQ). Eur J Pain. (2009) 13:620–8. 10.1016/j.ejpain.2008.11.001 19101182

[32] Kunz M Capito ES Horn-Hofmann C Baum C Scheel J Karmann AJ. Psychometric properties of the German version of the Pain Vigilance and Awareness Questionnaire (PVAQ) in pain-free samples and samples with acute and chronic pain. Int J Behav Med. (2017) 24:260–71. 10.1007/s12529-016-9585-4 27481106 PMC5344944

[33] Zigmond AS Snaith RP. The hospital anxiety and depression scale. Acta Psychiatr Scand. (1983) 67:361–70. 10.1111/j.1600-0447.1983.tb09716.x 6880820

[34] Albani C Blaser G Geyer M Schmutzer G Brähler E Bailer H. Überprüfung der Gütekriterien der deutschen Kurzform des Fragebogens “Profile of Mood States” (POMS) in einer repräsentativen Bevölkerungsstichprobe. Psychother Psychosom Med Psychol. (2005) 55:324–30. 10.1055/s-2004-834727 15986282

[35] Schulz U Schwarzer R. Soziale Unterstützung bei der Krankheitsbewältigung: Die Berliner Social Support Skalen (BSSS). [Social Support in Coping with Illness: The Berlin Social Support Scales (BSSS)]. Diagnostica. (2003) 49:73–82. 10.1026//0012-1924.49.2.73

[36] Bartholomew K Horowitz LM. Attachment styles among young adults: a test of a four-category model. J Pers Soc Psychol. (1991) 61:226–44. 10.1037/0022-3514.61.2.226 1920064

[37] Berryman C Stanton TR Bowering KJ Tabor A Mcfarlane A Moseley GL. Do people with chronic pain have impaired executive function? A meta-analytical review. Clin Psychol Rev. (2014) 34:563–79. 10.1016/j.cpr.2014.08.003 25265056

[38] Higgins DM Martin AM Baker DG Vasterling JJ Risbrough V. The relationship between chronic pain and neurocognitive function: a systematic review. Clin J Pain. (2018) 34:262–75. 10.1097/AJP.0000000000000536 28719507 PMC5771985

[39] Rodewald K Bartolovic M Debelak R Aschenbrenner S Weisbrod M Roesch-Ely D. Eine Normierungsstudie eines modifizierten Trail Making Tests im deutschsprachigen Raum. Zeitschrift für Neuropsychologie. (2012) 23:37–48. 10.1024/1016-264X/a000060

[40] Wagner S Helmreich I Dahmen N Lieb K Tadić A. Reliability of three alternate forms of the trail making tests A and B. Arch Clin Neuropsychol. (2011) 26:314–21. 10.1093/arclin/acr024 21576092

[41] Andraszewicz S Scheibehenne B Rieskamp J Grasman R Verhagen J Wagenmakers E-J. An introduction to bayesian hypothesis testing for management research. J Manage. (2014) 41:521–43. 10.1177/0149206314560412

[42] Smith SM Jenkinson M Woolrich MW Beckmann CF Behrens TEJ Johansen-Berg H. Advances in functional and structural MR image analysis and implementation as FSL. Neuroimage. (2004) 23:S208–19. 10.1016/j.neuroimage.2004.07.051 15501092

[43] Woolrich MW Jbabdi S Patenaude B Chappell M Makni S Behrens T. Bayesian analysis of neuroimaging data in FSL. Neuroimage. (2009) 45:S173–86. 10.1016/j.neuroimage.2008.10.055 19059349

[44] Jenkinson M Bannister P Brady M Smith S. Improved optimization for the robust and accurate linear registration and motion correction of brain images. Neuroimage. (2002) 17:825–41. 10.1006/nimg.2002.1132 12377157

[45] Smith SM. Fast robust automated brain extraction. Hum Brain Mapp. (2002) 17:143–55. 10.1002/hbm.10062 12391568 PMC6871816

[46] Beckmann CF Smith SM. Probabilistic independent component analysis for functional magnetic resonance imaging. IEEE Trans Med Imaging. (2004) 23:137–52. 10.1109/TMI.2003.822821 14964560

[47] Kelly RE Jr Alexopoulos GS Wang Z Gunning FM Murphy CF Morimoto SS. Visual inspection of independent components: defining a procedure for artifact removal from fMRI data. J Neurosci Methods. (2010) 189:233–45. 10.1016/j.jneumeth.2010.03.028 20381530 PMC3299198

[48] Cifre I Sitges C Fraiman D Muñoz MÁ Balenzuela P González-Roldán A. Disrupted functional connectivity of the pain network in fibromyalgia. Psychosom Med. (2012) 74:55–62. 10.1097/PSY.0b013e3182408f04 22210242

[49] Douaud G Mackay C Andersson J James S Quested D Ray MK. Schizophrenia delays and alters maturation of the brain in adolescence. Brain. (2009) 132:2437–48. 10.1093/brain/awp126 19477963

[50] Thomas AG Marrett S Saad ZS Ruff DA Martin A Bandettini PA. Functional but not structural changes associated with learning: an exploration of longitudinal voxel-based morphometry (VBM). Neuroimage. (2009) 48:117–25. 10.1016/j.neuroimage.2009.05.097 19520171 PMC2981435

[51] Petrovic P Petersson KM Ghatan PH Stone-Elander S Ingvar M. Pain-related cerebral activation is altered by a distracting cognitive task. Pain. (2000) 85. 10.1016/S0304-3959(99)00232-8 10692599

[52] Bantick SJ Wise RG Ploghaus A Clare S Smith SM Tracey I. Imaging how attention modulates pain in humans using functional MRI. Brain. (2002) 125:310–9. 10.1093/brain/awf022 11844731

[53] Villemure C Bushnell CM. Cognitive modulation of pain: how do attention and emotion influence pain processing? Pain. (2002) 95. 10.1016/S0304-3959(02)00007-6 11839418

[54] Moriarty O Mcguire BE Finn DP. The effect of pain on cognitive function: A review of clinical and preclinical research. Prog Neurobiol. (2011) 93:385–404. 10.1016/j.pneurobio.2011.01.002 21216272

[55] Eun S Lee J Song E-M Rosa AD Lee J-H Park K. Brain functional connectivity changes by low back extension pain model in low back pain patients. PLoS ONE. (2020) 15:e0233858. 10.1371/journal.pone.0233858 32479547 PMC7263586

[56] Buhle JT Kober H Ochsner KN Mende-Siedlecki P Weber J Hughes BL. Common representation of pain and negative emotion in the midbrain periaqueductal gray. Soc Cogn Affect Neurosci. (2013) 8:609–16. 10.1093/scan/nss038 22446299 PMC3739905

[57] Wiebking C Cellini C Wippert P-M. Reliable structural markers of depressed mood - preliminary findings of a longitudinal MRI study. In: 22nd Annual Meeting of the Organization for Human Brain Mapping. Genf, (2016).

[58] Grieve SM Korgaonkar MS Koslow SH Gordon E Williams LM. Widespread reductions in gray matter volume in depression. NeuroImage Clin. (2013) 3:332–9. 10.1016/j.nicl.2013.08.016 24273717 PMC3814952

