# Supplementary material for: Training intervention effects on cognitive performance and neuronal plasticity—A pilot study
Source: Front Neurol. 2022 Aug 5;13:773813. doi: 10.3389/fneur.2022.773813 (PMC9393784; doi:10.3389/fneur.2022.773813)
Supplement: Supplementary file 1 [file Data_Sheet_1.doc]

**Supplementary material**

**Analysis of MRI data**

Imaging data were processed using FSL (FMRIB Software Library, please also refer to https://fsl.fmrib.ox.ac.uk/fsl/fslwiki/FSL).

**Anatomical images**

- BET (Brain Extraction Tool): (Smith, 2002)⁠

- FAST (FMRIB's Automated Segmentation Tool): (Zhang et al., 2001)⁠

- FNIRT (FMRIB's Non-Linear Image Registration Tool): (Andersson et al., 2007)⁠

- Fslutils (please refer to https://fsl.fmrib.ox.ac.uk/fsl/fslwiki/Fslutils) such as

fslmaths (smoothing)

fslmerge (concatenation of images)

fslstats (summary statistics)

FAST segments a 3D image of the brain into different tissue types: grey matter, white matter, CSF, etc.). Grey matter images were used to quantify the proportion of grey matter located in regions of interest (see below). These values were used in correlation analyses (ROI to ROI, see 2.5.3) in order to control for the volume of grey matter within each region.

**Functional images**

- MCFLIRT (motion correction based on FLIRT) and FLIRT: (Jenkinson et al., 2002)⁠

- FSL Motion Outliers

- BET (Brain Extraction Tool): (Smith, 2002)⁠

- FSL Slice Timing Correction

- MELODIC (Multivariate Exploratory Linear Optimized Decomposition into Independent Components ) for registration, smoothing, filtering, ICA decomposition (Beckmann and Smith, 2004)⁠ and denoising (Kelly Jr. et al., 2010)⁠

- Fslutils such as fslmaths (definition of ROIs, see below)

- Atlases: Jülich histological atlas

Temporal signal courses within a target region and the remaining brain were performed using the Z-transformed correlation coefficients.

**Definition of ROIs**

Pain-related regions of interest (ROIs: periaqueductal grey (PAG), the primary motor cortex (M1), the primary/secondary somatosensory area (S1/S2) and the supplementary motor area (SMA) were defined as cubes of 3x3x3 voxels based on the paper by Cifre et al., 2012.

**M1** (ROI in blue): 10, -30, 70; -10, -26, 68 (Jülich histological atlas, red) (Cifre et al., 2012)⁠


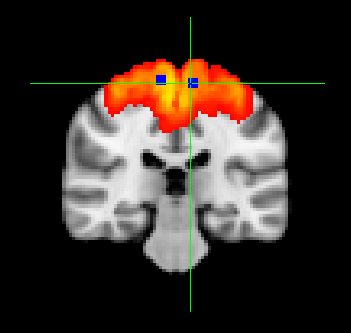

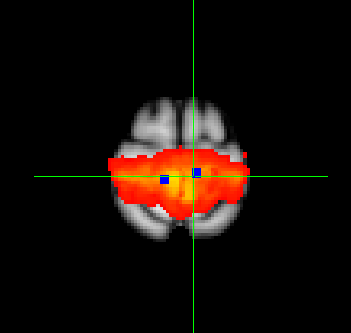

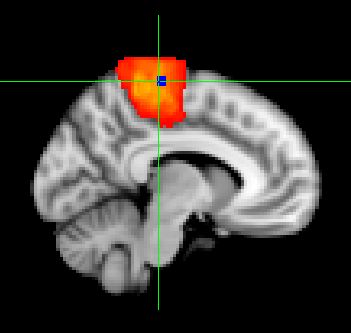


R

**PAG** (ROI in red): 6, -32, -10 (Zaki et al., 2007; Cifre et al., 2012)⁠; -6, -32, -10 (Cifre et al., 2012)
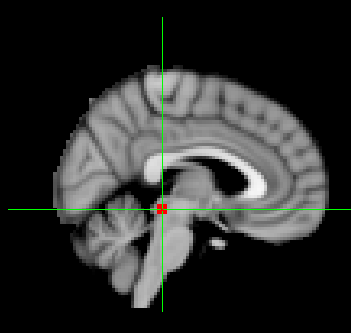
⁠


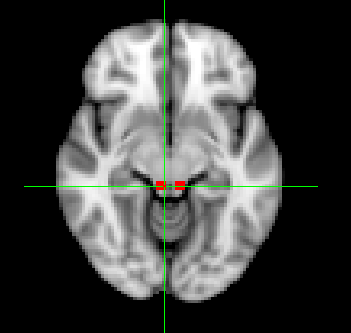

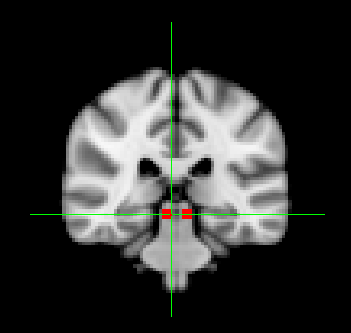


R

**SMA** (ROI in yellow): 12, 2, 68 (Gracely et al., 2002)⁠; -12, 2, 68 (Cifre et al., 2012)⁠


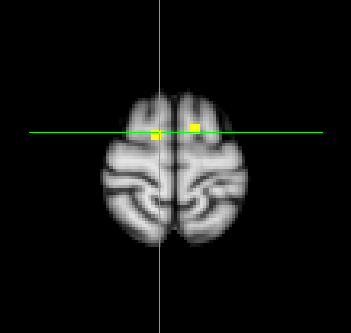

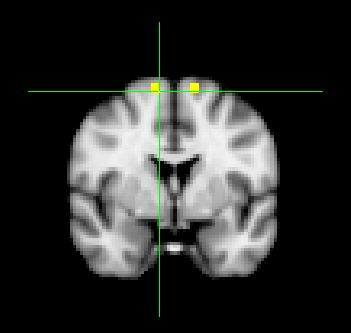

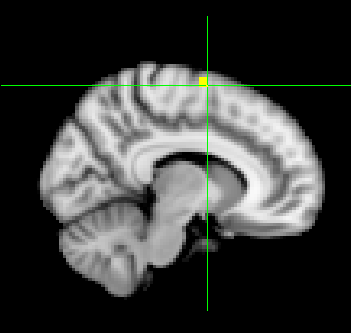


R

**S1** (ROI in green): 52, -16, 44 (Gracely et al., 2002; Cifre et al., 2012)⁠; -48, -24, 52 (Gracely et al., 2002; Cifre et al., 2012)⁠


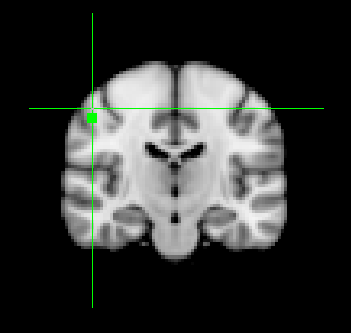

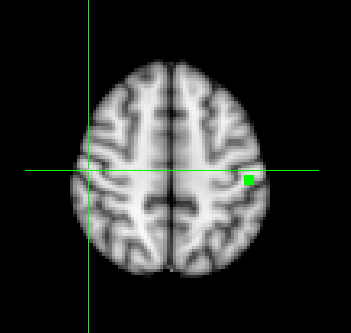

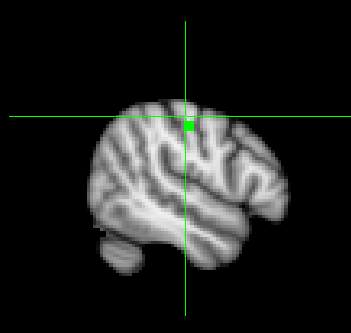


R

**S2** (ROI in red): 52, -20, 16 (Gracely et al., 2002; Cifre et al., 2012)⁠; -58, -24, 14 (Gracely et al., 2002; Cifre et al., 2012)⁠⁠


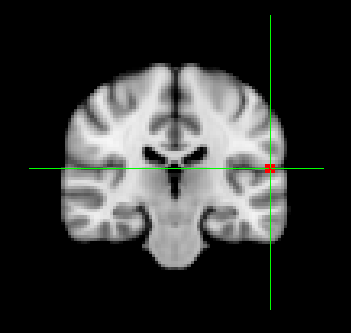

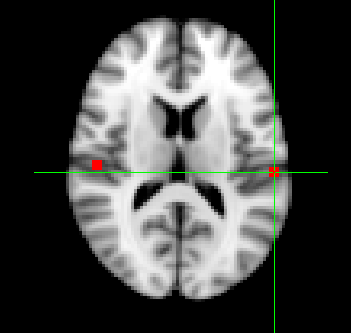

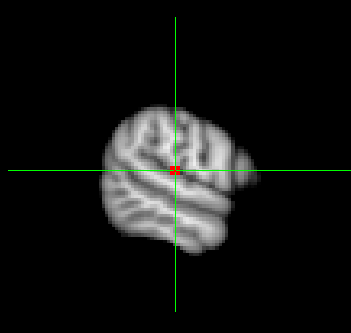


R

**References**

Andersson, J. L. R., Jenkinson, M., and Smith, S. (2007). Non-linear registration aka Spatial normalisation. FMRIB Technial Report TR07JA2 from www.fmrib.ox.ac.uk/analysis/techrep. Oxford, United Kingdom.

Beckmann, C. F., and Smith, S. M. (2004). Probabilistic independent component analysis for functional magnetic resonance imaging. *IEEE Trans Med Imaging* 23, 137–152. doi:10.1109/TMI.2003.822821.

Cifre, I., Sitges, C., Fraiman, D., Munoz, M. a., Balenzuela, P., Gonzalez-Roldan, a., Martinez-Jauand, M., Birbaumer, N., Chialvo, D. R., and Montoya, P. (2012). Disrupted Functional Connectivity of the Pain Network in Fibromyalgia. *Psychosom. Med.* 74, 55–62. doi:10.1097/PSY.0b013e3182408f04.

Gracely, R. H., Petzke, F., Wolf, J. M., and Clauw, D. J. (2002). Functional magnetic resonance imaging evidence of augmented pain processing in fibromyalgia. *Arthritis Rheum.* 46, 1333–1343. doi:10.1002/art.10225.

Jenkinson, M., Bannister, P., Brady, M., and Smith, S. (2002). Improved optimization for the robust and accurate linear registration and motion correction of brain images. *Neuroimage* 17, 825–41.

Kelly Jr., R. E., Alexopoulos, G. S., Wang, Z., Gunning, F. M., Murphy, C. F., Morimoto, S. S., Kanellopoulos, D., Jia, Z., Lim, K. O., Hoptman, M. J., et al. (2010). Visual inspection of independent components: defining a procedure for artifact removal from fMRI data. *J Neurosci Methods* 189, 233–245. doi:S0165-0270(10)00162-7 [pii] 10.1016/j.jneumeth.2010.03.028.

Smith, S. M. (2002). Fast robust automated brain extraction. *Hum Brain Mapp* 17, 143–155. doi:10.1002/hbm.10062.

Zaki, J., Ochsner, K. N., Hanelin, J., Wager, T. D., and Mackey, S. C. (2007). Different circuits for different pain: Patterns of functional connectivity reveal distinct networks for processing pain in self and others. *Soc Neur* 2, 276–291. doi:10.1080/17470910701401973.Different.

Zhang, Y., Brady, M., and Smith, S. (2001). Segmentation of brain MR images through a hidden Markov random field model and the expectation-maximization algorithm. *IEEE Trans. Med. Imaging* 20, 45–57. doi:10.1109/42.906424.
